# Admission lactate-to-albumin ratio in critically ill older adults: superior to its individual components but not additive to APACHE II — a retrospective cohort study

**DOI:** 10.1186/s12877-026-08495-3

**Published:** 2026-10-10

**Authors:** Seher Yanatma, Ahmet Düzgün

**Affiliations:** Department of Intensive Care, University of Health Sciences, Gazi Yaşargil Training and Research Hospital, Diyarbakır, Türkiye

**Keywords:** Lactate-to-albumin ratio, Critical illness, Older adults, Intensive care unit, 28-day mortality, APACHE II, Prognostic biomarker

## Abstract

**Background:**

Older adults constitute a growing and heterogeneous proportion of intensive care unit (ICU) admissions, and early risk stratification in this group remains difficult. The lactate-to-albumin ratio (LAR) is a composite biomarker that combines a marker of acute metabolic derangement with one of physiological reserve, and has shown prognostic value in several critical care settings. Its performance in geriatric ICU patients, and specifically whether it adds information to established severity scores, has not been established.

**Methods:**

We conducted a retrospective, single-centre cohort study of consecutive patients aged ≥ 65 years admitted to the mixed medical–surgical ICU of a tertiary referral hospital in Türkiye between January 2024 and July 2025. LAR was calculated as serum lactate (mmol/L) divided by serum albumin (g/dL), from samples obtained within the first 6 h of ICU admission. The primary outcome was all-cause 28-day mortality. Discrimination was assessed by area under the receiver operating characteristic curve (AUC), and curves were compared using the DeLong test. Multivariable logistic regression assessed whether LAR was independently associated with mortality after adjustment for age, sex, Clinical Frailty Scale (CFS), SOFA, APACHE II and C-reactive protein (CRP).

**Results:**

Of 296 screened admissions, 294 were analysed; 98 patients (33.3%) died within 28 days. Non-survivors had higher admission LAR than survivors [1.37 (IQR 0.84–2.50) vs. 0.62 (IQR 0.46–0.99), *p* < 0.001]. LAR discriminated 28-day mortality with an AUC of 0.793 (95% CI 0.740–0.846), significantly better than serum lactate alone (AUC 0.746; ΔAUC 0.047, 95% CI 0.021–0.074, *p* = 0.0005) and serum albumin alone (AUC 0.656; ΔAUC 0.137, 95% CI 0.061–0.213, *p* = 0.0004). APACHE II discriminated considerably better than LAR (AUC 0.924, 95% CI 0.893–0.955; ΔAUC 0.131, 95% CI 0.080–0.181, *p* < 0.001). Adding LAR to APACHE II did not significantly improve discrimination (AUC 0.929 vs. 0.924; ΔAUC 0.006, 95% CI −0.0001 to 0.0115, *p* = 0.055; likelihood ratio test *p* = 0.098). On univariable analysis LAR was strongly associated with mortality (OR 2.17, 95% CI 1.63–2.88, *p* < 0.001), but after full adjustment this association was no longer statistically significant (adjusted OR 1.29, 95% CI 1.00–1.66, *p* = 0.052). APACHE II (adjusted OR 1.36, 95% CI 1.21–1.52, *p* < 0.001) and CRP (adjusted OR 1.06 per 10 mg/L, 95% CI 1.01–1.11, *p* = 0.015) remained independently associated with mortality.

**Conclusions:**

In critically ill older adults, admission LAR discriminates 28-day mortality better than either of its components alone and is strongly associated with mortality on unadjusted analysis. However, it did not add significant discriminatory information to the APACHE II score, and it was not independently associated with mortality after adjustment for illness severity. LAR may be useful as a rapidly available early indicator where a full severity score is not yet computable, but on these data it does not complement APACHE II.

## Background

 The global population is rapidly aging, resulting in a marked increase in the number of geriatric patients admitted to intensive care units (ICUs) [1, 2]. Critically ill older adults represent a uniquely vulnerable population due to the higher prevalence of frailty, multimorbidity, impaired physiological reserve, and reduced resilience to acute stressors [3, 4]. Despite advances in critical care, this population exhibits considerable heterogeneity in outcomes; patients with comparable acute illness severity often experience markedly different clinical trajectories. This observation highlights that mortality risk in geriatric ICU patients is determined not only by the extent of acute physiological derangement but also by the individual’s underlying biological resilience and reserve.

Several validated prognostic scoring systems are used in clinical practice, with the Acute Physiology and Chronic Health Evaluation II (APACHE II) score remaining one of the most widely adopted tools worldwide [5]. APACHE II integrates acute physiological variables, age, and chronic health conditions to estimate mortality risk. However, its calculation is relatively complex and time-consuming, requiring multiple parameters that may not be immediately available upon admission. More importantly, it has been suggested that, while APACHE II performs adequately in general ICU populations, it may not fully capture the combined effects of systemic inflammation, metabolic stress, malnutrition, and frailty that are particularly prominent in older critically ill patients [6]. Accordingly, there is increasing interest in parsimonious biomarkers that may complement established severity scores by incorporating dimensions of biological vulnerability not fully represented in traditional prognostic models.

Serum lactate and albumin levels are routinely measured in critically ill patients and reflect complementary pathophysiological pathways [7, 8]. Elevated lactate indicates tissue hypoperfusion, cellular metabolic dysfunction, and ongoing stress response, whereas low albumin levels are associated with systemic inflammation, capillary leak, and impaired physiological reserve [9, 10]. The lactate-to-albumin ratio (LAR) integrates these two markers into a single, easily calculable parameter that may simultaneously reflect acute illness severity and the patient’s underlying biological vulnerability. In recent years, several studies have reported promising prognostic performance of LAR across various critical care settings, including sepsis, trauma, cardiac arrest, and mixed medical-surgical ICU cohorts [11–15].

Data specifically addressing the prognostic performance of LAR in geriatric ICU populations remain limited, and it is not established whether LAR adds information to severity scores such as APACHE II in this group. Given the particular characteristics of older critically ill patients — frailty, sarcopenia, chronic low-grade inflammation and reduced homeostatic capacity [16, 17] — a marker integrating metabolic stress and physiological reserve is conceptually attractive. We therefore evaluated the discriminative performance of admission LAR for 28-day mortality in geriatric ICU patients, compared it with its individual components and with APACHE II, and tested whether adding LAR to APACHE II improved mortality discrimination. We hypothesised that admission LAR would be independently associated with 28-day mortality and would provide incremental prognostic value beyond APACHE II.

## Methods

### Study design and setting

This was a retrospective, single-centre, observational cohort study conducted in the mixed medical–surgical adult ICU of a tertiary referral hospital in Türkiye. All consecutive patients aged 65 years and older admitted between January 2024 and July 2025 were screened for eligibility. The study protocol was approved by the Ethics Committee of Gazi Yaşargil Training and Research Hospital (approval number 244, date 05/06/2026) and was performed in accordance with the ethical standards of the Declaration of Helsinki. Because of the retrospective design and the use of anonymised routinely collected clinical data, the requirement for informed consent was waived. Reporting follows the STROBE statement for observational studies.

### Study population

Patients were eligible if they were aged ≥ 65 years and had admission serum lactate, serum albumin and an APACHE II score available, together with documented 28-day mortality status. Of 296 screened admissions, one was excluded because all clinical and laboratory fields were missing and one because the admission lactate value was not recorded, leaving 294 patients for analysis (Fig. 1). No patient had more than one ICU admission during the study period.

### Data collection

Data were extracted retrospectively from the hospital electronic medical record system and the departmental ICU database. Collected variables included demographics, comorbidities, the Charlson Comorbidity Index, admission Glasgow Coma Scale, Clinical Frailty Scale (CFS) [18], APACHE II and SOFA scores (calculated using the worst values within the first 24 h of ICU admission), admission laboratory parameters, and outcome variables.

The CFS was assigned retrospectively by chart review rather than prospectively at the bedside. For each patient, one investigator reviewed the ICU admission note and assigned a CFS score reflecting the patient’s baseline functional status in the two weeks preceding the acute illness, in accordance with the original scale definition. Because the assessment was performed by a single investigator, inter-rater agreement could not be calculated. Retrospective single-rater assignment of the CFS is a recognised source of misclassification and is addressed in the Limitations.

### Calculation of the lactate-to-albumin ratio

LAR was calculated as serum lactate (mmol/L) divided by serum albumin (g/dL). Serum albumin was measured and is reported throughout this manuscript in g/L, as issued by our laboratory; for the purpose of calculating LAR only, albumin values were converted to g/dL by dividing by 10, so that the ratio is expressed on the same scale as in the published literature. Serum lactate was measured by venous blood gas analysis, and serum albumin was measured on venous blood samples in the hospital’s central laboratory. All laboratory parameters were obtained within the first 6 h of ICU admission.

### Outcomes

The primary outcome was all-cause 28-day mortality after ICU admission. Secondary outcomes were the requirement for and duration of invasive mechanical ventilation, and ICU length of stay.

### Statistical analysis

Analyses were performed using IBM SPSS Statistics for Windows, version 28.0 (IBM Corp., Armonk, NY, USA). Comparison of ROC curves using the DeLong method was performed in R version 4.6.1 (R Foundation for Statistical Computing, Vienna, Austria) using the pROC package version 1.19.0.1 [19]. Continuous variables were assessed for normality using visual inspection and the Shapiro–Wilk test, and are presented as median (interquartile range, IQR); categorical variables are presented as n (%). Groups were compared using the Mann–Whitney U test for continuous variables and the χ² or Fisher exact test for categorical variables, as appropriate.

Discrimination for 28-day mortality was assessed by receiver operating characteristic (ROC) curve analysis for serum lactate, serum albumin, LAR and APACHE II [20]. Areas under the curve (AUC) are reported with 95% confidence intervals, and optimal cut-off values were determined using the Youden index. The combined model was constructed by entering APACHE II and LAR together as independent variables in a binary logistic regression model; the predicted probabilities from this model were saved and used as the test variable in a further ROC analysis. ROC curves were compared using the DeLong test, and differences in AUC (ΔAUC) are reported with 95% confidence intervals.

Univariable and multivariable logistic regression analyses were used to assess the association between LAR and 28-day mortality. The multivariable model included age, sex, CFS, SOFA, APACHE II and CRP, selected on the basis of clinical relevance and univariable association with mortality. Multicollinearity was assessed using the variance inflation factor (VIF), and model calibration using the Hosmer–Lemeshow test. The incremental contribution of LAR to a model containing APACHE II was assessed by likelihood ratio test. Adjusted odds ratios (ORs) are reported with 95% CIs. A two-sided p value < 0.05 was considered statistically significant.

## Results

### Study population

Of 296 screened ICU admissions of patients aged ≥ 65 years, 294 were included in the analysis (Fig. 1). Median age was 77 years (IQR 71–86) and 139 patients (47.3%) were male. Ninety-eight patients (33.3%) died within 28 days of ICU admission.

**Fig. 1:**
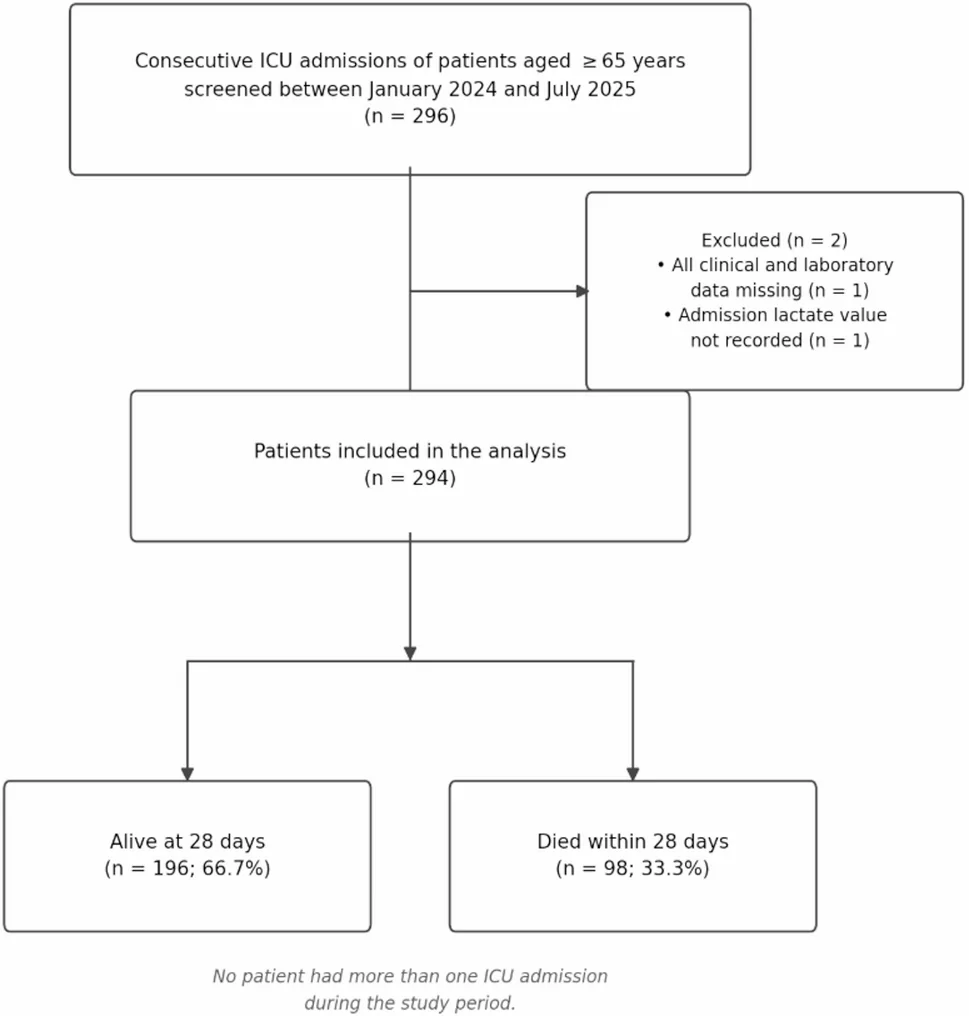
Study flow diagram. Of 296 screened ICU admissions of patients aged ≥ 65 years, one was excluded because all clinical and laboratory data were missing and one because the admission lactate value was not recorded, leaving 294 patients for analysis

### Baseline characteristics

Baseline demographic, clinical and laboratory characteristics according to 28-day mortality status are shown in Table 1. Age (*p* = 0.249) and sex distribution (*p* = 0.433) were comparable between survivors and non-survivors. Non-survivors had greater illness severity and comorbidity burden, with higher APACHE II [26 (IQR 23–31) vs. 14 (IQR 11–19), *p* < 0.001], SOFA [10 (IQR 9–12) vs. 6 (IQR 4–8), *p* < 0.001], CFS [7 (IQR 6–8) vs. 6 (IQR 5–7), *p* < 0.001] and Charlson Comorbidity Index scores [7 (IQR 6–8) vs. 6 (IQR 5–7), *p* < 0.001], and a lower admission Glasgow Coma Scale [6 (IQR 4–11) vs. 14 (IQR 11–15), *p* < 0.001].

**Table 1:** Baseline demographic, clinical and laboratory characteristics according to 28-day mortality status

Variable	All patients (*n* = 294)	Survivors (*n* = 196)	Non-survivors (*n* = 98)	*p*
Age, years	77 (71–86)	77 (71–85)	79 (71–87)	0.249
Male sex, n (%)	139 (47.3)	89 (45.4)	50 (51.0)	0.433
Charlson Comorbidity Index	6 (5–7)	6 (5–7)	7 (6–8)	< 0.001
Clinical Frailty Scale	6 (5–7)	6 (5–7)	7 (6–8)	< 0.001
Admission Glasgow Coma Scale	13 (8–14)	14 (11–15)	6 (4–11)	< 0.001
APACHE II score	19 (12–24)	14 (11–19)	26 (23–31)	< 0.001
SOFA score	8 (5–10)	6 (4–8)	10 (9–12)	< 0.001
Serum lactate, mmol/L	2.24 (1.58–3.65)	1.96 (1.44–2.83)	3.36 (2.11–8.04)	< 0.001
Serum albumin, g/L	30 (25–35)	31 (26–36)	26 (22–33)	< 0.001
Lactate-to-albumin ratio	0.78 (0.51–1.30)	0.62 (0.46–0.99)	1.37 (0.84–2.50)	< 0.001
C-reactive protein, mg/L	53.0 (11.4–110.6)	34.5 (7.9–84.3)	98.0 (24.0–163.8)	< 0.001

*Values are median (interquartile range) unless otherwise indicated. Serum albumin is reported in g/L as issued by the laboratory; for calculation of the lactate-to-albumin ratio only, albumin was converted to g/dL by dividing by 10. Continuous variables were compared with the Mann–Whitney U test and categorical variables with the χ² test. APACHE II, Acute Physiology and Chronic Health Evaluation II; SOFA, Sequential Organ Failure Assessment*

Non-survivors had higher admission serum lactate [3.36 (IQR 2.11–8.04) vs. 1.96 (IQR 1.44–2.83) mmol/L, *p* < 0.001], lower serum albumin [26 (IQR 22–33) vs. 31 (IQR 26–36) g/L, *p* < 0.001] and consequently a higher LAR [1.37 (IQR 0.84–2.50) vs. 0.62 (IQR 0.46–0.99), *p* < 0.001]. CRP was also higher in non-survivors [98.0 (IQR 24.0–163.8) vs. 34.5 (IQR 7.9–84.3) mg/L, *p* < 0.001].

### Discrimination for 28-day mortality

ROC analyses are presented in Table 2; Fig. 2. LAR discriminated 28-day mortality with an AUC of 0.793 (95% CI 0.740–0.846) at an optimal cut-off of 0.73 (sensitivity 82.7%, specificity 60.7%). LAR performed significantly better than either of its components alone: compared with serum lactate (AUC 0.746, 95% CI 0.687–0.805), ΔAUC was 0.047 (95% CI 0.021–0.074, *p* = 0.0005); compared with serum albumin (AUC 0.656, 95% CI 0.589–0.723), ΔAUC was 0.137 (95% CI 0.061–0.213, *p* = 0.0004).

**Table 2:** Discriminative performance for 28-day mortality and pairwise comparison of ROC curves

Parameter	AUC (95% CI)	Cut-off	Sens. (%)	Spec. (%)	Youden
APACHE II score	0.924 (0.893–0.955)	18.5	96.9	71.9	0.689
Lactate-to-albumin ratio	0.793 (0.740–0.846)	0.73	82.7	60.7	0.434
Serum lactate, mmol/L	0.746 (0.687–0.805)	4.06	45.9	89.8	0.357
Serum albumin, g/L	0.656 (0.589–0.723)	27.5	58.2	68.4	0.265
APACHE II + LAR	0.929 (0.899–0.960)	—	91.8	81.6	0.735
Pairwise comparison of ROC curves (DeLong test)					
Comparison	ΔAUC	95% CI	z	p	
APACHE II + LAR vs APACHE II	0.006	−0.0001 to 0.0115	1.92	0.055	
LAR vs serum lactate	0.047	0.021 to 0.074	3.50	0.0005	
LAR vs serum albumin	0.137	0.061 to 0.213	3.55	0.0004	
APACHE II vs LAR	0.131	0.080 to 0.181	5.07	<0.001	

*The APACHE II + LAR model was constructed by entering both variables into a binary logistic regression model; predicted probabilities from this model were used as the test variable in ROC analysis. This AUC was derived from the same data used to fit the model and is therefore an apparent, optimistically biased estimate. Cut-off values were determined by the Youden index. AUC, area under the receiver operating characteristic curve; CI, confidence interval; LAR, lactate-to-albumin ratio*

APACHE II showed markedly higher discrimination than LAR (AUC 0.924, 95% CI 0.893–0.955; ΔAUC 0.131, 95% CI 0.080–0.181, *p* < 0.001). The combination of APACHE II and LAR did not significantly improve discrimination over APACHE II alone (AUC 0.929, 95% CI 0.899–0.960; ΔAUC 0.006, 95% CI −0.0001 to 0.0115, *p* = 0.055).

### Association with 28-day mortality

On univariable logistic regression, LAR was strongly associated with 28-day mortality (OR 2.17 per unit increase, 95% CI 1.63–2.88, *p* < 0.001), as were APACHE II (OR 1.39, 95% CI 1.29–1.50), SOFA (OR 1.87, 95% CI 1.62–2.16), CFS (OR 1.60, 95% CI 1.34–1.92), the Charlson Comorbidity Index (OR 1.53, 95% CI 1.29–1.81) and CRP (OR 1.08 per 10 mg/L, 95% CI 1.05–1.12); all *p* < 0.001. Age (*p* = 0.186) and male sex (*p* = 0.364) were not associated with mortality.

**Fig. 2:**
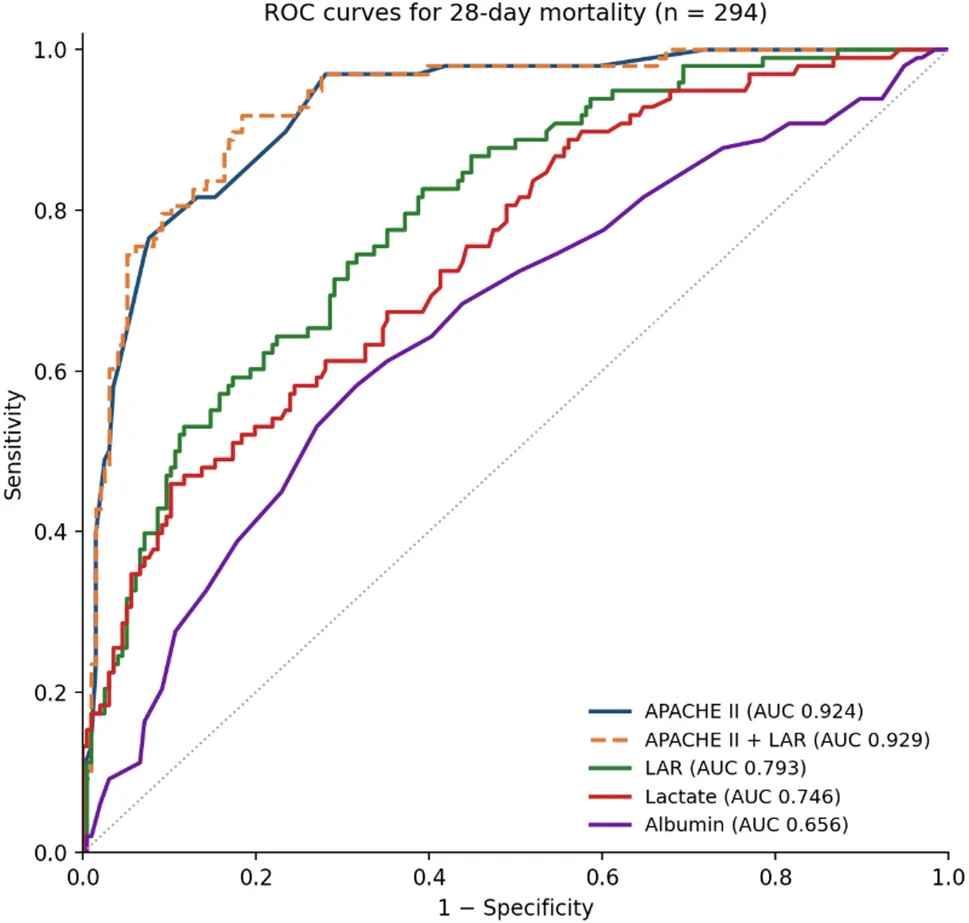
Receiver operating characteristic curves for the prediction of 28-day all-cause mortality. Curves compare the APACHE II score, the combination of APACHE II and the lactate-to-albumin ratio (LAR), LAR alone, serum lactate and serum albumin, in 294 critically ill patients aged ≥ 65 years. AUC values are shown in the legend

In the multivariable model (Table 3), APACHE II (adjusted OR 1.36, 95% CI 1.21–1.52, *p* < 0.001) and CRP (adjusted OR 1.06 per 10 mg/L, 95% CI 1.01–1.11, *p* = 0.015) remained independently associated with 28-day mortality. LAR was no longer statistically significant after adjustment (adjusted OR 1.29, 95% CI 1.00–1.66, *p* = 0.052), as were CFS (adjusted OR 0.91, *p* = 0.475) and SOFA (adjusted OR 1.03, *p* = 0.786). The addition of LAR to a model containing APACHE II did not significantly improve model fit (likelihood ratio χ²=2.74, df = 1, *p* = 0.098). The model explained a substantial proportion of the variance (Nagelkerke R²=0.666), but the Hosmer–Lemeshow test indicated suboptimal calibration (χ²=21.71, df = 8, *p* = 0.005). VIF values were 4.85 for APACHE II and 4.57 for SOFA, and below 1.6 for all other covariates, indicating moderate collinearity between the two severity scores.

**Table 3:** Multivariable logistic regression analysis for predictors of 28-day mortality

Variable	Adjusted OR	95% CI	*p*
Lactate-to-albumin ratio (per unit)	1.29	1.00–1.66	0.052
APACHE II score (per point)	1.36	1.21–1.52	< 0.001
C-reactive protein (per 10 mg/L)	1.06	1.01–1.11	0.015
Age (per year)	1.04	1.00–1.09	0.078
Male sex	1.55	0.72–3.34	0.264
Clinical Frailty Scale (per point)	0.91	0.69–1.19	0.475
SOFA score (per point)	1.03	0.81–1.31	0.786

*Model performance: Nagelkerke R² = 0.666; Hosmer–Lemeshow χ² = 21.71, df = 8, p = 0.005. Variance inflation factors: APACHE II 4.85, SOFA 4.57, Clinical Frailty Scale 1.51, lactate-to-albumin ratio 1.26, C-reactive protein 1.13, age 1.12, sex 1.07. CI, confidence interval; OR, odds ratio*

In models that did not contain APACHE II, LAR remained independently associated with mortality (with age, sex and CFS: adjusted OR 2.07, 95% CI 1.55–2.76, *p* < 0.001; with the additional inclusion of SOFA: adjusted OR 1.42, 95% CI 1.11–1.83, *p* = 0.006), indicating that the loss of statistical significance in the full model is attributable to the strong discriminative performance of APACHE II.

### Secondary outcomes

Secondary outcomes are summarised in Table 4. All 98 non-survivors required invasive mechanical ventilation at some point during their ICU stay, compared with 68 of 196 survivors (34.7%, *p* < 0.001); this near-universal requirement among non-survivors probably reflects intubation during terminal deterioration rather than a prognostic association. The duration of invasive mechanical ventilation was longer in non-survivors [4 (IQR 2–9) vs. 0 (IQR 0–5) days, *p* < 0.001]. ICU length of stay did not differ significantly between groups [6 (IQR 2–14) vs. 6 (IQR 2–19) days, *p* = 0.326], which is consistent with early deaths shortening ICU stay among non-survivors.

**Table 4:** Secondary outcomes according to 28-day mortality status

Outcome	Survivors (*n* = 196)	Non-survivors (*n* = 98)	*p*
Invasive mechanical ventilation required, n (%)	68 (34.7)	98 (100)	< 0.001
Duration of invasive mechanical ventilation, days	0 (0–5)	4 (2–9)	< 0.001
ICU length of stay, days	6 (2–19)	6 (2–14)	0.326

*Values are median (interquartile range) unless otherwise indicated. Continuous variables were compared with the Mann–Whitney U test and categorical variables with the χ² test. The near-universal requirement for invasive ventilation among non-survivors most probably reflects intubation during terminal deterioration rather than a prognostic association. ICU, intensive care unit*

## Discussion

In this retrospective cohort of 294 critically ill patients aged ≥ 65 years, admission LAR discriminated 28-day mortality moderately well and significantly better than either serum lactate or serum albumin alone. However, LAR discriminated considerably less well than APACHE II, adding LAR to APACHE II did not significantly improve discrimination, and LAR was not independently associated with mortality after adjustment for illness severity. Our a priori hypothesis that LAR would provide incremental prognostic value beyond APACHE II was therefore not supported.

The observation that LAR outperforms its individual components is biologically coherent. Elevated lactate reflects tissue hypoperfusion and metabolic stress [7], whereas hypoalbuminaemia reflects systemic inflammation, capillary leak and diminished physiological reserve [9, 10]. Combining the two captures both dimensions and, in our cohort, yielded a statistically significant gain in discrimination over each component (ΔAUC 0.047 and 0.137, respectively). In older patients, in whom frailty, sarcopenia and chronic low-grade inflammation are prevalent [16, 17], the albumin component may add information about physiological reserve that lactate alone does not capture [21]. At its optimal cut-off, LAR was considerably more sensitive than specific (82.7% versus 60.7%), a profile more consistent with use as an early screening indicator than as a confirmatory test.

Our findings are broadly consistent with the literature in showing an association between higher LAR and adverse outcomes across a range of clinical settings. Bou Chebl et al. reported that LAR independently predicted in-hospital mortality in septic patients presenting to the emergency department and outperformed lactate alone [11]; Erdogan and Findikli found similar results in patients with pneumosepsis [12]; Liu et al. in acute pancreatitis [13]; and Wang et al. in acute myocardial infarction admitted to the ICU [14]. More recently, Ağaçkıran and Ağaçkıran demonstrated the prognostic utility of LAR for ICU admission and in-hospital mortality in patients presenting with upper gastrointestinal bleeding [22], and reported comparable findings for LAR and the albumin-corrected anion gap in methanol poisoning [23]. Across these heterogeneous settings LAR performs consistently as a marker of risk, and our data extend this observation to an exclusively geriatric ICU population [24, 25]. Reported effect sizes nonetheless vary considerably: in an unselected cohort of more than 6,000 ICU patients, LAR discriminated ICU mortality only marginally better than lactate alone (AUC 0.69 versus 0.67) [7], suggesting that the advantage of the ratio over its components may be modest in heterogeneous populations.

Where our findings diverge from much of the existing literature is on incremental value. Most prior studies compared LAR with its components, with SOFA, or with disease-specific scores, rather than with a comprehensive severity score such as APACHE II. In our cohort APACHE II achieved an AUC of 0.924, and against this benchmark the additional contribution of LAR was small (ΔAUC 0.006) and did not reach statistical significance (*p* = 0.055). This is a coherent finding rather than a contradiction: APACHE II already incorporates acute physiological derangement, age and chronic health status, and a two-variable ratio is unlikely to add substantially once such a score is available. Notably, LAR did remain independently associated with mortality in models that excluded APACHE II, which supports this interpretation.

The discriminative performance of APACHE II in our cohort was nonetheless higher than is commonly reported for this score in general ICU populations [5, 6], and this deserves comment. Several factors may contribute. APACHE II was calculated from the worst values recorded during the first 24 h and therefore incorporates physiological deterioration occurring after the admission LAR sample was drawn. Our cohort also spanned a wide range of illness severity, with little overlap in APACHE II scores between survivors and non-survivors. In addition, severity scores generally perform better in the single-centre cohorts in which they are evaluated than in external validation studies. The absence of incremental value for LAR should therefore be interpreted in the context of an unusually strong comparator, and may not generalise to settings in which APACHE II discriminates closer to its usual range.

An important methodological consideration is the difference in the timing of the variables compared. LAR was derived from samples obtained within the first 6 h of ICU admission, whereas APACHE II and SOFA were calculated from the worst values within the first 24 h. This favours the severity scores, which incorporate information accumulated over a longer period, including deterioration that occurred after the LAR sample was drawn. The comparison is therefore not entirely symmetrical, and the incremental value of LAR may have been underestimated. Conversely, this asymmetry reflects the practical clinical question: LAR is available within minutes of admission, whereas a complete APACHE II score is not.

Contrary to our expectation and to several previous reports [18, 26, 27], CFS was not independently associated with 28-day mortality in the fully adjusted model, despite a strong univariable association (OR 1.60, 95% CI 1.34–1.92, *p* < 0.001). The point estimate in the adjusted model fell below unity (adjusted OR 0.91), but with a confidence interval spanning 1.00 (0.69–1.19) this is most consistent with the absence of an independent effect rather than with a protective one. The most likely explanation is that the acute severity scores included in the model, particularly APACHE II, capture much of the same prognostic information in this cohort. Retrospective assignment of the CFS may also have attenuated the association through misclassification.

Strengths of this study include an exclusively geriatric cohort of moderate size, adjustment for both established severity scores and frailty, formal statistical comparison of ROC curves with confidence intervals for ΔAUC, the use of parameters that are available immediately at admission, and the near-complete capture of eligible admissions, with only two of 296 screened patients excluded for missing data.

### Limitations

This study has several limitations. It was conducted in a single centre and was retrospective in design, which limits generalisability and leaves open the possibility of unmeasured confounding and selection bias.

A number of limitations relate to measurement. The timing of the variables compared was not uniform: LAR was based on values obtained within 6 h of admission, whereas APACHE II and SOFA used the worst values within 24 h. As discussed above, this asymmetry favours the severity scores and may have led to underestimation of the incremental value of LAR. Lactate was measured on venous rather than arterial blood; venous concentrations are on average slightly higher than arterial values, and since most previous studies of LAR have used arterial measurements, the cut-off reported here may not be directly transferable to settings in which arterial sampling is routine. The Clinical Frailty Scale was assigned retrospectively from the ICU admission note alone, by a single investigator and without a second independent rater, so inter-rater reliability could not be assessed; retrospective single-rater assignment is subject to misclassification, which would tend to bias the estimated association between frailty and mortality towards the null.

Further limitations concern the analysis. The study was not powered for a prespecified effect size; with 98 events and seven covariates the multivariable model approached the conventional limit of ten events per variable, and the borderline result for LAR (*p* = 0.052) may reflect limited statistical power rather than the absence of an association. The AUC of the combined APACHE II + LAR model was derived from the same data used to fit the model and is therefore an apparent, optimistically biased estimate, as no internal validation by bootstrapping or cross-validation was performed. The fully adjusted model showed suboptimal calibration (Hosmer–Lemeshow *p* = 0.005), and moderate collinearity was present between APACHE II and SOFA (VIF 4.85 and 4.57), which may have contributed to the loss of statistical significance of SOFA and CFS.

With respect to outcomes, ICU and in-hospital mortality were not available as separate variables, and the universal requirement for invasive mechanical ventilation among non-survivors most probably reflects terminal intubation rather than a prognostic relationship. We also did not evaluate whether LAR-guided management strategies alter patient outcomes.

## Conclusions

In critically ill older adults, the admission lactate-to-albumin ratio discriminates 28-day mortality better than either serum lactate or serum albumin alone and is strongly associated with mortality on unadjusted analysis. It did not, however, add significant discriminatory information to the APACHE II score, and it was not independently associated with mortality after adjustment for illness severity. LAR may have a role as an immediately available early indicator of risk in the first hours of ICU admission, before a full severity score can be computed, but our data do not support its use as a complement to APACHE II. Prospective multicentre studies using contemporaneous measurement windows, together with formal assessment of clinical utility using decision curve analysis, are needed to define its place in geriatric critical care.

## Data Availability

The datasets generated and analysed during the current study are not publicly available because they contain information that could compromise patient privacy, but are available from the corresponding author on reasonable request and subject to approval by the institutional ethics committee.

## Abbreviations

APACHE II: Acute Physiology and Chronic Health Evaluation II
AUC: Area under the receiver operating characteristic curve
CFS: Clinical Frailty Scale
CI: Confidence interval
CRP: C-reactive protein
GCS: Glasgow Coma Scale
ICU: Intensive care unit
IQR: Interquartile range
LAR: Lactate-to-albumin ratio
OR: Odds ratio
ROC: Receiver operating characteristic
SOFA: Sequential Organ Failure Assessment
STROBE: Strengthening the Reporting of Observational Studies in Epidemiology
VIF: Variance inflation factor
